# Burden, patterns, and impact of multimorbidity in North India: findings from a rural population-based study

**DOI:** 10.1186/s12889-022-13495-0

**Published:** 2022-06-02

**Authors:** Priti Gupta, Shivani A. Patel, Hanspria Sharma, Prashant Jarhyan, Rakshit Sharma, Dorairaj Prabhakaran, Nikhil Tandon, Sailesh Mohan

**Affiliations:** 1 Centre for Chronic Disease Control, New Delhi, India; 2 Emory University, Atlanta, GA USA; 3 All India Institute of Medical Sciences, New Delhi, India; 4 Public Health Foundation of India, Gurgaon, Haryana India; 5 Deakin University, Melbourne, Australia

**Keywords:** Multimorbidity, Multiple long term conditions, Multiple chronic conditions, Burden, Self-rated heath, Mental health, Activities of daily living, Depression, India

## Abstract

**Aim:**

To estimate the prevalence, socio-demographic determinants, common disease combinations, and health impact of multimorbidity among a young rural population.

**Methods:**

We conducted a cross-sectional survey among participants aged ≥30 years in rural Punjab, North India, from Jan 2019 to April 2019. Multimorbidity was defined as the coexistence of ≥two conditions using a 14-condition tool validated in India. We also calculated a multimorbidity-weighted index (MWI), which provides a weight to each disease based on its impact on physical functioning. Logistic regression was conducted to evaluate the association with sociodemographic variables, mental health (PHQ-9), physical functioning (ADL scale), and self-rated health (SRH).

**Results:**

We analyzed data from 3213 adults [Mean age 51.5 (±13), 54% women]. Prevalence of single chronic condition, multimorbidity, and MWI was 28.6, 18% and − 1.9 respectively. Age, higher wealth index and ever use alcohol were significantly associated with multimorbidity. Overall, 2.8% of respondents had limited physical functioning, 2.1% had depression, and 61.5% reported low SRH. Poorer health outcomes were more prevalent among the elderly, women, less educated, and those having lower wealth index and multimorbidity, were found to be significantly associated with poor health outcomes.

**Conclusions:**

The burden of multimorbidity was high in this young rural population, which portends significant adverse effects on their health and quality of life. The Indian health system should be reconfigured to address this emerging health priority holistically, by adopting a more integrated and sustainable model of care.

**Supplementary Information:**

The online version contains supplementary material available at 10.1186/s12889-022-13495-0.

## Background

With increased longevity and the rising burden of chronic non-communicable diseases (NCDs), multimorbidity or “multiple long term conditions” is emerging as a high priority public health concern globally. Considered to be more prevalent among the elderly, multimorbidity has notably started affecting younger age groups in low-middle income countries (LMICs) [1, 2]. Yet most data are derived from studies of older adults residing in high-income countries (HICs). This data gap is particularly stark in India, where several population-based studies show that chronic conditions are rapidly rising [3]. Available data indicate a multimorbidity prevalence between 30 and 83% among the elderly in India [3–5] and 5–45% [6, 7] among the young/middle-age population. This wide variation in the prevalence estimates is attributable to varied definitions of multimorbidity, methods of data collection, number of chronic diseases included in the analysis, age of the participants, and study settings (rural, urban, and primary health care). In addition, there is limited use of validated tools to measure the occurrence of multimorbidity in population-based studies [8]. Nonetheless, given that most NCDs are escalating in India and their onset is a decade earlier in comparison to HICs, it is likely that multimorbidity is also high [9].

Furthermore, multimorbidity also adversely affects an individual’s psychosocial wellbeing and daily functioning. A systematic review concluded that multimorbidity can predict a future decline in activities of daily living [10]. Data from the WHO study on global AGEing and adult health (SAGE) from six LMICs highlights that disability, poor self-rated health, and depression increases and quality of life decreases, with the number of co-occurring conditions [9]. In addition to determining multimorbidity’s association with various determinants, understanding the specific disease combinations or patterns of disease-causing disability, depression, and quality of life may help inform the development of suitable interventions for patients. However, limited studies from India report the effect of multimorbidity on mental health, functional well-being, and self-rated health.

Thus, there is an urgent need to conduct well designed studies to determine the burden of multimorbidity and its various dimensions, especially among the young, so that appropriate prevention and control measures can be designed, implemented and evaluated. The three main objectives of this study were: 1) to measure the prevalence of multimorbidity and its association with socio-demographic variables; 2) to determine the common disease combinations; 3) to examine the association of multimorbidity with mental health (depressive symptoms), self-rated overall health, and physical functioning (activities of daily living), among participants aged ≥30 years in rural Punjab, North India**.**

## Methods

### Study design and population

The study was conducted in Mukandpur and Sujjon block of Shaheed Bhagat Singh Nagar district of Punjab, North India from Jan 2019 to April 2019. The data were collected as part of the baseline community assessment in the context of a larger implementation science study to evaluate a technology-enabled platform to improve the diagnosis and management of hypertension and diabetes in the public healthcare system in India. The technology-enabled platform was embedded within the National Programme for Prevention and Control of Cancer, Diabetes, Cardiovascular Diseases and Stroke (NPCDCS), the flagship NCD screening and management program by the Government of India. Following the NPCDCS NCD screening guidelines, we targeted adults ages 30 years and older in the baseline community assessment [11,12].

The community assessment was a cross-sectional survey with a multistage sampling design. Households were selected using systematic random sampling from village lists created by field workers. From each household, two participants aged ≥30 years were randomly selected using the Kish method [11]. Pregnant women and bedridden participants were excluded. Three repeated house visits were made to contact the selected participants.

The community assessment included over 3000 adults. Post hoc power calculations show that 750 participants were needed to estimate a multimorbidity prevalence of 20% with 80% power, alpha of 0.05, a 2% margin of error, considering a response rate of 80% and a design effect of 1.5 [13]. Therefore, the current analysis had sufficient statistical power to estimate multimorbidity prevalence with desired precision.

### Data collection

Interviews were conducted by eight trained field workers at the home of the participants using a mobile data collection application with pre-coded skips and cross-checks to ensure data quality. Prior to the field activity, survey tools and procedures were pilot-tested and in-depth training provided to field workers. Field workers had the prior experience of collecting data in the field, were from the same community, and most of them had nursing qualification.

### Chronic conditions and multimorbidity

The presence of 14 conditions was assessed in the population using an adapted version of the Multimorbidity Assessment Questionnaire for Primary Care, which was developed and previously validated in India [14]. The adapted instrument was used to evaluate the presence of the following conditions: hypertension, diabetes, heart disease, stroke, hearing problem, thyroid disease, chronic kidney disease (CKD), chronic liver disease (CLD), low back pain, arthritis, chronic obstructive pulmonary disease (COPD), epilepsy, cancer, and tuberculosis (TB).

Participants were asked about prior medical diagnosis (hypertension, diabetes, heart disease, thyroid disease, COPD, cancer, CLD, stroke, arthritis) or current/past treatment (TB, CKD) for each condition. For epilepsy, low back pain, stroke, and hearing problem, symptom-related questions were asked (Refer Appendix A for details).

The self-reported diseases were verified with corresponding patient-held medical records, when available with the participant. The patient-held medical records included consultation, diagnostic and treatment documentation.

### Multimorbidity weighted index

Multimorbidity was defined as the coexistence of two or more conditions. As the simple disease count measure fails to capture the effect of diseases on current and future functional status [15], we also calculated a multimorbidity-weighted index (MWI), which weights each disease by its impact on current and future physical functioning and mortality. Weights for each disease were calculated as change in Short Form 36 physical functioning (PF) scale [16] over a period, using data from the US nationally representative prospective studies. The range of MWI is from − 9.11 to 0. Minus sign denotes a decrease in physical functioning score over a time period [15, 17]. Weights of 14 conditions included in present study were: hypertension (− 1.53), diabetes (− 2.67), arthritis (− 3.52), chronic obstructive pulmonary disease (− 1.62), heart disease (− 2.20), paralysis (− 3.79), CKD (− 3.98), alcohol liver disease (− 0.293), low back pain (− 1.46), tuberculosis (− 1.46) deafness (− 1.46), cancer(− 1.76), epilepsy (− 0.841), thyroid (− 0.808). The final multimorbidity weight was the weighted sum of all conditions reported (i.e., an additive method). For example, a person with both hypertension and diabetes will be assigned a final multimorbdity weight of − 4.2 [− 1.53+ (− 2.67)].

#### Mental health

The Patient Health Questionnaire (PHQ-9) was used to assess the presence of depressive symptoms. Depressive symptoms were dichotomized as high (PHQ-9 score ≥ 10) and low (PHQ-9 score < 10) [18].

### Physical functioning

Limitation in activities of daily living (ADL) was used to assess physical functioning. The questions were based on self-reported difficulty in engaging in activities using the Barthel Activity of Daily Living (ADL) scale. Physical functioning was dichotomized as limited physical functioning (ADL < 20) or no limitations in physical functioning (ADL = 20) [19].

### Self-rated health (SRH)

Participants were asked to rate “how good your health is today, in your opinion” on a scale of 0–100% (0 means worst and 100% means the best). The question is comparable to the sixth question of the EuroQoL-5 Dimension questionnaire (EQ-5D) published in 1990 [20] and has been widely used in previous literature as a measure of objective health and predictors of mortality [21, 22]. SRH was dichotomized as good (score > 80; median score) and poor (≤80) [21].

### Socio-demographic and health risk factors

As potential individual-level risk factors for multimorbidity, we examined sociodemographic factors (participant-reported age, gender, marital status, education, employment status, and occupation), behavioral risk factors (tobacco and alcohol use, physical inactivity), and basic anthropometry data (height and weight). Ever use of tobacco and alcohol was also assessed. Recommended level of physical activity was defined as walking/engaging in sport for at least 30 minutes a day on five days a week.

We categorized education level (up to primary, high school or secondary, college graduation and above); and occupation (professional/medium and big business owner, skilled labourer/small business, unskilled/semiskilled, homemaker). For calculating the wealth index, we used an index of household income, 35 amenities and assets. We used principal components analysis methods [23] and categorized the wealth index in tertiles.

### Statistical analysis

We estimated the prevalence and 95% CI of single morbidity and multimorbidity for the whole sample and across socio-demographic and health risk factors. For multimorbidity weight calculation, multimorbidity-weighted index (MWI) using the additive model was used [17]. Associations of a three-level variable classifying all participants as having either no morbidities, single morbidity, or multimorbidity with socio-demographic variables and health outcomes (i.e. mental health, physical functioning, and self-rated health) were estimated using the multinominal logistic regression model. All models were adjusted for all socio-demographic variables collected in the study. We have used adjusted regression analysis to assess the association of MWI with socio-demographic variables.

A simple matrix approach was used to determine the patterns (dyads and triads) of different multimorbidity combinations. We identified and reported all possible combinations of two or three chronic conditions [24]. Prevalence for a single chronic condition, combinations of two (dyad) and three chronic conditions (triad) were calculated for the whole study sample. In addition to identify different combination, we have also done cluster analysis to identify clusters of disease. For this, we have used agglomerative hierarchical clustering using Jaccard coefficients [25]. and obtained clusters based on a cluster dendrogram [26].

We evaluated the association of number of chronic conditions with mental health, physical functioning, and self-rated health using a separate multinominal regression models for each outcome. Outcome variables for this analysis were no disease, single disease, two disease and ≥ 3 chronic disease. All models were adjusted for demographic and chronic disease risk factors.

### Ethics approval

The study protocol was approved by the Institutional ethics committee of the Centre for Chronic Disease Control, New Delhi. Written informed consent was obtained from all the participants.

## Results

### Sample characteristics, the prevalence of multimorbidity, and other health outcomes

We analyzed data from 3213 adults (54% women). Table 1 presents the percentage distribution and 95% CI of socioeconomic and demographic characteristics of the study population and the prevalence of single morbidity, multimorbidity, and mean multimorbidity weighted index (MWI). The overall prevalence of having at least one chronic condition out of the 14 listed conditions was 28.6%, prevalence of multimorbidity was 18% and MWI was − 1.9. Prevalence of single morbidity among the young (< 45 years), middle-aged (45–59 years) and elderly (> 60 years) was 25, 30, and 30.8% respectively; and the prevalence of multimorbidity was 8.5, 17.4, 29.8% respectively.

**Table 1:** Prevalence of multimorbidity and mean multimorbidity weighted index by socio-demographic characteristics

Study participants characteristics		Prevalence of morbidity		
		Single morbidity (*n* = 920)	Multimorbidity (*n* = 579)	Multimorbidity weighted Index (MWI)
Total (*n* = 3213)	100.0	28.6 [27.1–30.2]	18.0 [16.7–19.4]	− 1.9 [1.94,-1.8]
**Age**	% [95% CI]	% [95% CI]	% [95% CI]	% [95% CI]
30–44 years (*n* = 1055)	32.6 [31.0–34.3]	25.1 [22.5–27.8]	8.5 [6.9–10.3]	− 1.1[− 1.2,-1.02]
45–59 years (*n* = 1259)	39 [37.3–40.7]	30.0 [27.6–32.6]	17.4 [15.4–19.6]	− 1.9 [− 2.0,-1.76]
60 years and above (*n* = 912)	28.4 [26.9–30.0]	30.8 [27.9–33.9]	29.8 [26.9–32.9]	− 2.7 [− 2.89,-2.53]
**Gender**				
Men (*n* = 1478)	46.0 [44.3–47.7]	24.1 [22.0–26.3]	15.9 [14.1–17.9]	− 1.7 [− 1.82,-1.58]
Women (*n* = 1735)	54.0 [52.3–55.7]	32.5 [30.3–34.7]	19.8 [18.0–21.8]	− 2.0 [− 2.11,-1.91]
**Marital status**				
Currently married (*n* = 2748)	85.5 [84.3–86.7]	29.4 [27.7–31.1]	17.0 [15.7–18.5]	− 1.8 [− 1.91,-1.74]
Widow/Widower/ separated / divorced (*n* = 399)/	12.4 [11.3–13.6]	26.6 [22.5–31.1]	26.8 [22.7–31.4]	− 2.4 [− 2.68,-2.13]
Never married (*n* = 66)	2.1 [1.6–2.6]	9.1 [4.1–18.8]	6.1 [2.3–15.1]	−0.7 [− 1.03,-0.36]
**Education status**				
College graduation and above (*n* = 147)	4.6 [3.9–5.4]	36.1 [28.7–44.1]	12.2 [7.9–18.6]	− 1.7 [− 1.99,-1.33]
High school or secondary (*n* = 2219)	69.1 [67.4–70.6]	27.4 [25.6–29.3]	15.8 [14.3–17.4]	− 1.7 [− 1.79,-1.62]
Up to primary (*n* = 847)	26.4 [24.9–27.9]	30.5 [27.5–33.6]	24.9 [22.1–27.9]	− 2.3 [− 2.53,-2.17]
**Occupation**				
Professional/medium and big business owner (*n* = 166)	5.8 [5.0–6.7]	34.3 [27.5–41.9]	12.7 [8.4–18.6]	− 1.7 [− 2.00,− 1.41]
Skilled labourer/small business (*n* = 314)	10.9 [9.8–12.1]	24.5 [20.1–29.6]	11.5 [8.4–15.5]	-1.4 [− 1.64,-1.19]
Unskilled/semiskilled (*n* = 910)	31.6 [29.9–33.3]	23.0 [20.3–25.8]	9.5 [7.7–11.5]	− 1.3 [− 1.37,-1.13]
Homemaker (*n* = 1490)	51.7 [49.9–53.6]	31.9 [29.6–34.4]	21.7 [19.7–23.8]	− 2.1 [− 2.24,-2.01]
**Wealth index**				
Low (*n* = 1143)	35.6 [34.0–37.3]	24.3 [21.9–26.9]	15.9 [13.9–18.2]	− 1.6 [− 1.70,-1.45]
Medium (*n* = 1015)	31.6 [30.1–33.3]	29.0 [26.3–31.8]	17.0 [14.9–19.5]	− 1.9 [− 2.05,-1.77]
High (*n* = 1050)	32.7 [31.1–34.4]	33.0 [30.2–35.9]	21.3 [19.0–23.9]	− 2.2 [− 2.31,-2.03]
**Physical activity**				
Yes (*n* = 2692)	83.8 [82.5–85.0]	28.4 [26.7–30.1]	17.0 [15.6–18.5]	− 1.8 [− 1.91,-1.74]
No (*n* = 521)	16.2 [15.0–17.5]	29.9 [26.2–34.0]	23.2 [19.8–27.0]	− 2.1 [− 2.33,-1.92]
**Ever alcohol use**				
Yes (*n* = 814)	25.3 [23.9–26.9]	22.6 [19.9–25.6]	18.3 [15.8–21.1]	− 1.8 [− 1.97,-1.63]
No (*n* = 2399)	74.7 [73.1–76.1]	30.7 [28.9–32.6]	17.9 [16.4–19.5]	− 1.9 [− 1.99,-1.81]
**Ever tobacco use**				
Yes (*n* = 481)	15.0 [13.8–16.2]	21.6 [18.2–25.5]	16.0 [13.0–19.6]	− 1.6 [− 1.85,-1.42]
No (*n* = 2732)	85.0 [83.8–86.2]	29.9 [28.2–31.6]	18.4 [17.0–19.9]	− 1.9 [− 2.00,− 1.83]
**BMI**^**#**^				
Under-weight (*n* = 171)	5.3 [4.6–6.2]	22.8 [17.1–29.7]	18.7 [13.5–25.3]	-1.8 [− 2.13,-1.40]
Normal (*n* = 640)	19.9 [18.6–21.3]	24.2 [21.1–27.7]	15.6 [13.0–18.7]	− 1.6 [− 1.80,-1.47]
Overweight (*n* = 469)	14.6 [13.4–15.9]	29.6 [25.7–33.9]	13.9 [11.0–17.3]	− 1.6 [− 1.79,-1.43]
Obese (*n* = 1933)	60.2 [58.5–61.8]	30.4 [28.4–32.5]	19.8 [18.0–21.6]	− 2.0 [− 2.13,-1.92]
**Abdominal obesity**^**a**^				
Normal (*n* = 830)	25.8 [24.3–27.4]	23.6 [20.8–26.6]	10.8 [8.9–13.1]	− 1.3 [− 1.38,-1.13]
Central obesity (*n* = 2383)	74.2 [72.6–75.7]	30.4 [28.6–32.3]	20.5 [18.9–22.2]	− 2.1 [− 2.18,-2.00]

^a^Used South Asian cut offs for BMI i.e. BMI 23–24.9 kg/m2 for overweight and ≥ 25 kg/m2 for obesity, Waist circumference ≥ 80 cm for women and ≥ 90 cm for men for abdominal obesity [23]

Table 2 presents the findings of adjusted multinomial logistic regression models used to examine the association of sociodemographic, and health risk factors with multimorbidity. The ‘no disease’ category was the reference group in the multinomial logit regression model. There was a positive association between age group and both single and multimorbidity. Compared with men, women were significantly more likely to have single morbidity than no disease, whereas gender was not significantly associated with multimorbidity. Higher wealth index, obesity, and alcohol use had a positive association with multimorbidity. Similar to count of disease measure, MWI was also significantly associated with age, higher wealth index and obesity (Supplementary Table 5).

**Table 2:** Multinomial logit model estimates examining the association of multimorbidity with socio-demographic variables

Variables	One disease versus no disease		Multimorbidity versus no disease	
	AOR (95%CI)	*P* value	AOR (95%CI)	*P* value
Age category (Ref-30-44 years)				
45–59 years	1.49 [1.2–1.8]	< 0.001	2.41 [1.8–3.2]	< 0.001
60 years and above	1.88 [1.5–2.4]	< 0.001	4.28 [3.1–6.0]	< 0.001
Gender (Ref- Male)	1.43 [1.1–1.9]	< 0.001	1.25 [0.8–1.9]	0.3
Marital status (Ref: Never married)				
Currently married	3.10 [1.2–8.2]	< 0.001	1.68 [0.5–5.6]	0.4
Widow/widower	2.26 [0.8–6.2]	0.1	1.60 [0.5–5.6]	0.4
Separated / divorced	1.00 [0.1–9.0]	1.0	3.35 [0.4–25.2]	0.2
Education (Ref: high school)				
College graduation and above	1.36 [0.9–2.1]	0.2	0.81 [0.4–1.6]	0.5
Up to primary	1.23 [1.0–1.5]	0.1	1.26 [1.0–1.7]	0.2
Occupation (Ref: unskilled/semiskilled)				
Professional/medium and big business	1.45 [1.0–2.2]	0.1	1.41 [0.8–2.5]	0.3
Skilled labourer/small business	1.11 [0.8–1.5]	0.5	1.22 [0.8–1.9]	0.4
Homemaker	1.44 [1.1–1.9]	< 0.001	3.29 [2.2–4.9]	< 0.001
Wealth Index (Ref: Low)				
Medium	1.24 [1.0–1.6]	0.1	1.12 [0.8–1.5]	0.5
High	1.41 [1.1–1.8]	< 0.001	1.43 [1.1–1.9]	< 0.001
Physical activity				
Yes	1.22 [0.9–1.6]	0.1	1.27 [0.9–1.8]	0.1
Ever alcohol use				
Yes	0.88 [0.6–1.2]	0.4	1.51 [1.0–2.2]	< 0.001
Ever tobacco use				
Yes	1.08 [0.8–1.5]	0.6	1.29 [0.9–2.0]	0.3
BMI^a^				
Under-weight	1.10 [0.7, 1.8]	0.6	0.81 [0.4–1.5]	0.6
Overweight	1.29 [0.9–1.8]	0.1	0.99 [0.7–1.5]	0.9
Obese	1.41 [1.1–1.8]	< 0.001	1.63 [1.2–2.2]	< 0.001

^a^Used south Asian cut off for BMI i.e. BMI 23–24.9 kg/m2 for overweight and ≥ 25 kg/m2 for obesity [23]

Table 3 displays the prevalence of three health outcomes across socio-demographic variables and morbidity status. Overall, 2.8% of respondents had 1+ ADL limitation, 2.1% had depression, and 61.5% reported low SRH. Prevalence of disability, depression, and low self-rated health is highest among participants with > 2 chronic conditions i.e. 12.2 (CI: 8.3–17.6); 5.6 (CI: 3.1–9.9); 91.3 (CI: 86.5–94.5), respectively. After adjustment with socio-demographic variables, poorer health outcomes were generally more prevalent among the elderly, women, less educated, lower wealth index less physically active and have more chronic conditions (Supplementary Table 4).

**Table 3:** Prevalence (95% Confidence Intervals) of health outcomes by socio-demographic characteristics and morbidity status

	Disability (*n* = 89)	Depression (*n* = 68)	Low self-rated health (*n* = 155)
Total (*n* = 3213)	2.8 [2.3–3.4]	2.1 [1.7–2.7]	61.5 [59.8–63.1]
Age	% [95% CI]	% [95% CI]	% [95% CI]
30–44 years (n = 1055)	0.5 [0.2–1.1]	1.8 [1.2–2.8]	44.1 [41.2–47.2]
45–59 years (*n* = 1259)	1.4 [0.9–2.3]	1.7 [1.1–2.6]	63.5 [60.8–66.1]
≥60 years (*n* = 912)	7.2 [5.7–9.1]	3.1 [2.1–4.4]	78.6 [75.8–81.2]
Gender			
Men (*n* = 1478)	2.4 [1.7–3.3]	0.9 [0.6–1.6]	53.3 [50.8–55.8]
Women (*n* = 1735)	3.1 [2.4–4.0]	3.1 [2.4–4.0]	68.4 [66.2–70.6]
Marital status			
Currently Married (*n* = 2748)	2.1 [1.6–2.7]	1.9 [1.4–2.4]	59.9 [58.1–61.7]
Widow/Widower/ separated / divorced (*n* = 399)	7.8 [5.5–10.8]	4.0 [2.5–6.4]	76.2 [71.8–80.1]
Never Married (*n* = 66)	1.5 [0.2–10.0]	1.5 [0.2–10.0]	37.9 [27.0–50.1]
Education status			
College graduation and above (*n* = 147)	0.7 [0.1–4.7]	0.0	44.9 [37.1–53.0]
High school or secondary (*n* = 2219)	1.8 [1.4–2.5]	1.4 [0.9–1.9]	56 [53.9–58.0]
Up to primary (*n* = 847)	5.5 [4.2–7.3]	4.5 [3.3–6.1]	78.7 [75.9–81.4]
Occupation			
Professional/Medium and big business owner (*n* = 166)	0.6 [0.1–4.2]	0.0	46.4 [38.9–54.0]
Skilled labourer/small business (*n* = 314)	0.3 [0.0–2.2]	0.6 [0.2–2.5]	43 [37.6–48.5]
Unskilled/semiskilled (*n* = 910)	0.5 [0.2–1.3]	1.5 [0.9–2.6]	51.5 [48.3–54.8]
Homemaker (*n* = 1490)	2.1 [1.5–2.9]	2.3 [1.7–3.3]	68.1 [65.7–70.4]
Wealth index			
Low (*n* = 1143)	2.7 [1.9–3.8]	3.6 [2.7–4.8]	65.4 [62.6–68.1]
Medium (*n* = 1015)	3.2 [2.2–4.4]	1.8 [1.1–2.8]	59.4 [56.4–62.4]
High (*n* = 1050)	2.5 [1.7–3.6]	0.7 [0.3–1.4]	59 [56.0–62.0]
Physical activity			
Yes (*n* = 2692)	1.9 [1.4–2.4]	1.7 [1.3–2.3]	59.2 [57.3–61.0]
No (*n* = 521)	7.5 [5.5–10.1]	4.2 [2.8–6.3]	73.3 [69.4–76.9]
Ever alcohol use			
Yes (*n* = 814)	2.5 [1.6–3.8]	1.0 [0.5–2.0]	54.9 [51.5–58.3]
No (*n* = 2399)	2.9 [2.3–3.6]	2.5 [1.9–3.2]	63.7 [61.7–65.6]
Ever tobacco use			
Yes (*n* = 481)	2.9 [1.7–4.9]	1.5 [0.7–3.0]	60.9 [56.5–65.2]
No (*n* = 2732)	2.7 [2.2–3.4]	2.2 [1.7–2.9]	61.6 [59.7–63.4]
BMI			
Under-weight (*n* = 171)	8.2 [4.9–13.4]	5.8 [3.2–10.5]	71.3 [64.1–77.6]
Normal (*n* = 640)	3.8 [2.5–5.5]	2.2 [1.3–3.7]	61.6 [57.7–65.3]
Overweight (*n* = 469)	1.9 [1.0–3.6]	2.3 [1.3–4.2]	57.6 [53.0–62.0]
Obese (*n* = 1933)	2.2 [1.6–2.9]	1.7 [1.2–2.4]	61.5 [59.3–63.7]
Abdominal obesity			
Normal (*n* = 830)	3.3 [2.2–4.7]	3.1 [2.1–4.6]	58.3 [54.9–61.6]
Central obesity (*n* = 2383)	2.6 [2.0–3.3]	1.8 [1.3–2.4]	62.6 [60.6–64.5]
No of morbidities			
0 (*n* = 1714)	0.9 (0.6–1.5)	1.5 (1.0–2.2)	51.1 (48.7–53.5)
1 (*n* = 920)	3 (2.1–4.4)	1.8 (1.2–3.0)	65.0 (61.9–68.0)
2 (*n* = 383)	5.5 (3.6–8.3)	3.7 (2.2–6.1)	84.1 (80.1–87.4)
3 or more (*n* = 196)	12.2 (8.3–17.6)	5.6 (3.1–9.9)	91.3 (86.5–94.5)

The adjusted association between multimorbidity and depression, physical functioning, and SRH, are presented in Table 4. Compared with adults with no disease, the relative odds of having limited physical functioning, depression, and self-rated health increases around three-fold in participants with two or more chronic diseases. The count of chronic diseases was positively associated with limited physical functioning, poor mental, and self-rated health. Compared to adults with no disease, having three or more diseases was associated with five times the relative odds of limited physical functioning [5.2(CI: 1.9–14.1)], depression [4.2 (CI: 1.5–12.0)], and low SRH [5.2 (CI: 3.1, 9)].

**Table 4:** Impact of multimorbidity on participant’s mental health, physical functioning, and self-rated health (SRH)

Health outcomes	One disease versus no disease		Two disease versus no disease		≥ 3 disease versus no disease	
	AOR (95% CI)	*P* value	AOR (95% CI)	*P* value	AOR (95% CI)	*P* value
Disability	1.9 (0.8, 4.6)	0.2	2.2 (0.8, 6.1)	0.1	5.2 (1.9, 14.1)	0.001
Depression	1.1 (0.5, 2.4)	0.8	2.1 (0.9, 5.2)	0.1	4.2 (1.5, 12)	0.007
SRH	1.4 (1.2, 1.7)	< 0.001	3.4 (2.5, 4.6)	< 0.001	5.2 (3.1, 9)	< 0.001
Health outcomes	One disease versus no disease		Multimorbidity versus no disease			
	AOR (95% CI)	*P* value	AOR (95% CI)	*P* value		
Disability	1.9 (0.8, 4.6)	0.2	3.2 (1.4, 7.8)	0.007		
Depression	1.1 (0.5, 2.4)	0.8	2.7 (1.2, 6.2)	0.01		
SRH	1.4 (1.2, 1.7)	< 0.001	3.8 (2.8, 5.0)	< 0.001		

### Different combinations of chronic conditions

The prevalence of the 14 chronic conditions has been provided in a Supplementary Table 1 in the supplementary file. In total, 920 (28%) of the participants had single chronic conditions. The most prevalent single chronic condition either in isolation or in combinations were hypertension (20%), low back pain (18.9%), diabetes (9.2%), and arthritis (8.4%). The most common dyads were hypertension and diabetes, most common triad was hypertension arthritis and low back pain [Supplementary Tables 2 and 3].

**Fig. 1:**
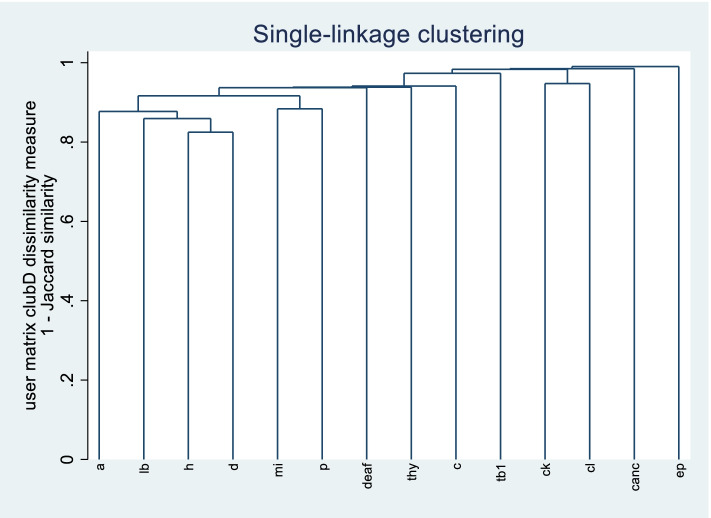
Cluster dendrogram for Jaccard dissimilarity coefficient. Note: a = arthritis, lb. = lower back pain, h = hypertension, d = diabetes, mi = myocardial infarction, p = paralysis, deaf = deafness, thy = thyroid disorder, c = COPD, tb = Tuberculosis, ck = CKD, cl = alcoholic liver disease, canc = cancer, ep = epilepsy

Clusters analysis: Clusters were identified using cluster dendrogram. In dendrogram diseases which join together sooner are more similar to each other than those that joins later. Hypertension and diabetes had the smallest normed flexible distance and formed the first cluster to appear in the dendrogram. Myocardial infarction and paralysis were the second cluster. Chronic kidney disease and alcoholic liver disease were the third cluster. Therefore, three clusters identified were i.e. 1) hypertension, diabetes; 2) Myocardial infarction and paralysis 3) Chronic kidney disease and alcoholic liver disease (Fig. 1).

## Discussion

In this sample of young adults, more than one-fourth had at least one chronic disease and around one-fifth had multimorbidity. A third and quarter of the elderly had a single morbidity and multimorbidity respectively, while 8.5% of those aged under 45 had multimorbidity, indicating a high burden of both single and multimorbidity. While single morbidity and multimorbidity both increase with age, the gradient across age groups was steeper for multimorbidity in higher age groups. Chances of having multimorbidity was also higher among participants belonging to higher wealth index, having history of ever use alcohol and obesity. Hypertension, low back pain, diabetes, and arthritis were found to be most common chronic conditions. Hypertension and diabetes are found to be most common conditions which occur together. Multimorbidity was found to be associated with participants’ poor physical functioning, mental and self-rated health.

Previous multimorbidity studies from Punjab show that the prevalence among the elderly is around 35% [27], while that among those aged ≥45 years is 4.7% [28]. A recent study from Kerala among aged 30 years and above has reported the prevalence of multimorbidity to be 45% [7], but in this study participants with raised blood pressure diagnosed at the time of the survey were included as hypertensive. In our study also, if we had included individuals with newly diagnosed hypertension and diabetes, the prevalence of multimorbidity would have risen to around 45%. The reason we did not include participants with raised blood pressure measured during the study as hypertensive was because previous studies have reported that the use of single blood pressure measurements to diagnose hypertension can lead to misclassification and the use of multiple readings at different time points reduces the prevalence of hypertension to 12% and almost 35% of patients are reclassified as normotensive [29–31]. In another community-based study from India, the prevalence of multimorbidity was 9.1% [32] among adults aged ≥20 years. However, this study included a lesser number of diseases, and participants were younger than our study population. The prevalence of multimorbidity among elderly aged ≥60 years was 30%, which is similar to the results reported in earlier studies conducted in the rural elderly in India [4, 27]. Other studies from Indian rural populations also had reported a higher prevalence of multimorbidity [33, 34]. In summary, true comparison from different studies is difficult due to inconsistency in the definitions, inclusion of different age groups, and different settings. However, based on our study and other almost similar studies from India, we can infer that the burden is high and it is increasing even among the young rural population across the country, in a milieu of increasing NCDs. The odds of having limited physical functioning, depression, and poor self-rated health was higher with increases in the number of diseases which has been reported from other studies in India, other LMICs and HICs [28, 35, 36].

The high burden of multimorbidity among rural young (i.e. ≥30 years) is disconcerting for the constrained Indian health system which is still orientated towards provision of maternal and child care and services for communicable diseases, using auxiliary nurse midwifes (ANMs) and accredited social health activists (ASHAs), who are the backbone of Indian Primary Health Care system., They also have very limited capacity for addressing NCDs. In addition, there is a paucity of qualified MBBS doctors (allopathic doctors) in rural India and primary health centres (PHCs) are often managed by AYUSH (non-allopathic alternative system) physicians [37]. Therefore, to address multimorbidity there is a more patient-centered and integrated model of care. The planned Health and Wellness Centres under Ayushman Bharat Program that aims to reform primary care has the potential to provide more comprehensive primary health care and provides an opportunity to address multimorbidity more holistically [38].

Most of the previous studies conducted in India were conducted among the elderly and in Southern India. In this large study, we have included an extensive list of 14 chronic conditions which covers majors NCDs and chronic infections in comparison to earlier studies based on 6–10 chronic diseases. Further, we have also assessed the impacts of multimorbidity on adult psychosocial and physical functioning. However, there are few limitations. Firstly, the severity index of individual diseases was not included, and results are based on the disease count measures. To account for this we have used previously validated multimorbidity weights and results are almost similar to disease count measures. Secondly, we have ascertained multimorbidity through self-reports which are subject to recall bias and interviewer bias. However, the questionnaire used is well validated and used in previous studies from India [14, 39], and we have done extensive training of field workers to arrive at the correct diagnosis. Despite this, ascertainment of acid peptic disease and vision problems had few challenges in the field and we have dropped them from our analysis. Similarly, we also dropped filariasis and dementia considering the challenges in its assessment in the field. Thirdly, since this study was part of a larger study in which bed-ridden participants were excluded, the prevalence of disability is likely to be underreported in our study population. Fourth, all associations may not have been detected due to small sample size in some demographic strata.

## Conclusion

Using standardized methods and measurements, this study provides contemporary evidence of increasing multimorbidity in a rural setting. Despite the growing burden, multimorbidity has not received adequate recognition from health care providers and policymakers alike. The health system is still focused on individual disease management rather than having an integrated care model. The present COVID-19 pandemic has further highlighted the importance of multimorbidity in increasing the severity of COVID-19 and attendant mortality. Thus, this study’s findings underline the need for the development and implementation of an integrated and sustainable model of care to prevent and manage multimorbidity. Furthermore, there is a need to conduct well designed prospective studies to determine incidence, common clusters, health and economic impacts.

## Supplementary Information

**Additional file 1.**

## Data Availability

The data used for this study is available with the corresponding author and can be shared upon reasonable request.
