# Supplementary material for: Burden, patterns, and impact of multimorbidity in North India: findings from a rural population-based study
Source: BMC Public Health. 2022 Jun 2;22:1101. doi: 10.1186/s12889-022-13495-0 (PMC9159928; doi:10.1186/s12889-022-13495-0)
Supplement: Supplementary file 1 — Additional file 1. [file 12889_2022_13495_MOESM1_ESM.docx]

**Appendix A**

- For epilepsy, question asked was “Have you ever suffered from a sudden onset of seizure while at work or rest?”
- For low back pain; question asked was “In the last 12 months, have you had continuous back pain for more than three weeks?”
- For stroke question asked was “In the last 12 months have you suffered from a sudden onset of paralysis or weakness in your arms or legs on one side of your body for more than 24 hours?”
- For hearing problem question asked was: “Do you have difficulty in hearing?”

**Supplementary table 1: Prevalence of diseases**

| Prevalence of diseases (in isolation or combination) | | | | | | | | | | | | |
| --- | --- | --- | --- | --- | --- | --- | --- | --- | --- | --- | --- | --- |
|  | Gender | | | | Age | | | | | | Overall | |
|  | Male | | Female | | 30-44years | | 45-59 years | | 60 years and above | |  |  |
|  | Col % | 95% CI | Col % | 95% CI | Col % | 95% CI | Col % | 95% CI | Col % | 95% CI | Col % | 95% CI |
| Hypertension |  |  |  |  |  |  |  |  |  |  |  |  |
| 0 (n=2,564) | 82.4 | [80.4,84.3] | 77.6 | [75.6,79.5] | 90.2 | [88.2,91.8] | 79.1 | [76.7,81.2] | 68.9 | [65.8,71.8] | 79.8 | [78.4,81.2] |
| 1 (n=649) | 17.6 | [15.7,19.6] | 22.4 | [20.5,24.4] | 9.8 | [8.2,11.8] | 20.9 | [18.8,23.3] | 31.1 | [28.2,34.2] | 20.2 | [18.8,21.6] |
| Total (n=3,213) | 100 |  | 100 |  | 100 |  | 100 |  | 100 |  | 100 |  |
|  |  |  |  |  |  |  |  |  |  |  |  |  |
| Low back pain |  |  |  |  |  |  |  |  |  |  |  |  |
| 0 (n=2,605) | 86.5 | [84.7,88.2] | 76.4 | [74.4,78.4] | 81.3 | [78.8,83.6] | 82.1 | [79.9,84.1] | 79.4 | [76.6,81.9] | 81.1 | [79.7,82.4] |
| 1 (n=608) | 13.5 | [11.8,15.3] | 23.6 | [21.6,25.6] | 18.7 | [16.4,21.2] | 17.9 | [15.9,20.1] | 20.6 | [18.1,23.4] | 18.9 | [17.6,20.3] |
| Total (n=3,213) | 100 |  | 100 |  | 100 |  | 100 |  | 100 |  | 100 |  |
|  |  |  |  |  |  |  |  |  |  |  |  |  |
| Diabetes |  |  |  |  |  |  |  |  |  |  |  |  |
| 0 (n=2,916) | 91 | [89.4,92.4] | 90.6 | [89.1,91.8] | 97.4 | [96.3,98.2] | 90.2 | [88.4,91.7] | 83.9 | [81.3,86.1] | 90.8 | [89.7,91.7] |
| 1 (n=297) | 9 | [7.6,10.6] | 9.4 | [8.2,10.9] | 2.6 | [1.8,3.7] | 9.8 | [8.3,11.6] | 16.1 | [13.9,18.7] | 9.2 | [8.3,10.3] |
| Total (n=3,213) | 100 |  | 100 |  | 100 |  | 100 |  | 100 |  | 100 |  |
|  |  |  |  |  |  |  |  |  |  |  |  |  |
| Arthritis |  |  |  |  |  |  |  |  |  |  |  |  |
| 0 (n=2,944) | 93 | [91.6,94.2] | 90.4 | [89.0,91.7] | 96.4 | [95.1,97.4] | 91.9 | [90.2,93.2] | 85.9 | [83.4,88.0] | 91.6 | [90.6,92.5] |
| 1 (n=269) | 7 | [5.8,8.4] | 9.6 | [8.3,11.0] | 3.6 | [2.6,4.9] | 8.1 | [6.8,9.8] | 14.1 | [12.0,16.6] | 8.4 | [7.5,9.4] |
| Total (n=3,213) | 100 |  | 100 |  | 100 |  | 100 |  | 100 |  | 100 |  |
|  |  |  |  |  |  |  |  |  |  |  |  |  |
| Thyroid Disease |  |  |  |  |  |  |  |  |  |  |  |  |
| 0 (n=3,088) | 98.4 | [97.6,98.9] | 94.2 | [93.0,95.2] | 95.1 | [93.7,96.3] | 95.8 | [94.5,96.8] | 97.7 | [96.5,98.5] | 96.1 | [95.4,96.7] |
| 1 (n=125) | 1.6 | [1.1,2.4] | 5.8 | [4.8,7.0] | 4.9 | [3.7,6.3] | 4.2 | [3.2,5.5] | 2.3 | [1.5,3.5] | 3.9 | [3.3,4.6] |
| Total (n=3,213) | 100 |  | 100 |  | 100 |  | 100 |  | 100 |  | 100 |  |
|  |  |  |  |  |  |  |  |  |  |  |  |  |
| Heart disease (HD) | |  |  |  |  |  |  |  |  |  |  |  |
| 0 (n=3,095) | 95.7 | [94.5,96.6] | 96.9 | [96.0,97.6] | 99 | [98.1,99.4] | 96.5 | [95.3,97.4] | 93.1 | [91.3,94.6] | 96.3 | [95.6,96.9] |
| 1 (n=118) | 4.3 | [3.4,5.5] | 3.1 | [2.4,4.0] | 1 | [0.6,1.9] | 3.5 | [2.6,4.7] | 6.9 | [5.4,8.7] | 3.7 | [3.1,4.4] |
| Total (n=3,213) | 100 |  | 100 |  | 100 |  | 100 |  | 100 |  | 100 |  |
|  |  |  |  |  |  |  |  |  |  |  |  |  |
| Deafness |  |  |  |  |  |  |  |  |  |  |  |  |
| 0 (n=3,111) | 95.8 | [94.7,96.7] | 97.7 | [96.9,98.3] | 99.5 | [98.9,99.8] | 98.2 | [97.3,98.8] | 91.8 | [89.8,93.4] | 96.8 | [96.2,97.4] |
| 1 (n=102) | 4.2 | [3.3,5.3] | 2.3 | [1.7,3.1] | 0.5 | [0.2,1.1] | 1.8 | [1.2,2.7] | 8.2 | [6.6,10.2] | 3.2 | [2.6,3.8] |
| Total (n=3,213) | 100 |  | 100 |  | 100 |  | 100 |  | 100 |  | 100 |  |
|  |  |  |  |  |  |  |  |  |  |  |  |  |
| COPD |  |  |  |  |  |  |  |  |  |  |  |  |
| 0 (n=3,157) | 98.3 | [97.5,98.9] | 98.2 | [97.5,98.7] | 99.8 | [99.2,100.0] | 98.3 | [97.4,98.9] | 96.4 | [95.0,97.4] | 98.3 | [97.7,98.7] |
| 1 (n=56) | 1.7 | [1.1,2.5] | 1.8 | [1.3,2.5] | 0.2 | [0.0,0.8] | 1.7 | [1.1,2.6] | 3.6 | [2.6,5.0] | 1.7 | [1.3,2.3] |
| Total (n=3,213) | 100 |  | 100 |  | 100 |  | 100 |  | 100 |  | 100 |  |
|  |  |  |  |  |  |  |  |  |  |  |  |  |
| Stroke |  |  |  |  |  |  |  |  |  |  |  |  |
| 0 (n=3,158) | 97.2 | [96.3,98.0] | 99.2 | [98.6,99.5] | 99.1 | [98.4,99.6] | 98.4 | [97.5,99.0] | 97.1 | [95.8,98.1] | 98.3 | [97.8,98.7] |
| 1 (n=55) | 2.8 | [2.0,3.7] | 0.8 | [0.5,1.4] | 0.9 | [0.4,1.6] | 1.6 | [1.0,2.5] | 2.9 | [1.9,4.2] | 1.7 | [1.3,2.2] |
| Total (n=3,213) | 100 |  | 100 |  | 100 |  | 100 |  | 100 |  | 100 |  |
|  |  |  |  |  |  |  |  |  |  |  |  |  |
| Tuberculosis |  |  |  |  |  |  |  |  |  |  |  |  |
| 0 (n=3,192) | 99.2 | [98.6,99.5] | 99.5 | [99.0,99.7] | 99.3 | [98.6,99.7] | 99.4 | [98.8,99.7] | 99.2 | [98.4,99.6] | 99.3 | [99.0,99.6] |
| 1 (n=21) | 0.8 | [0.5,1.4] | 0.5 | [0.3,1.0] | 0.7 | [0.3,1.4] | 0.6 | [0.3,1.2] | 0.8 | [0.4,1.6] | 0.7 | [0.4,1.0] |
| Total (n=3,213) | 100 |  | 100 |  | 100 |  | 100 |  | 100 |  | 100 |  |
|  |  |  |  |  |  |  |  |  |  |  |  |  |
| Chronic Liver Disease | |  |  |  |  |  |  |  |  |  |  |  |
| 0 (n=3,198) | 99 | [98.3,99.4] | 100 | 99.5 | 99.5 | [98.9,99.8] | 99.4 | [98.8,99.7] | 99.7 | [99.0,99.9] | 99.5 | [99.2,99.7] |
| 1 (n=15) | 1 | [0.6,1.7] | 0 | 0.5 | 0.5 | [0.2,1.1] | 0.6 | [0.3,1.2] | 0.3 | [0.1,1.0] | 0.5 | [0.3,0.8] |
| Total (n=3,213) | 100 |  | 100 | 100 | 100 |  | 100 |  | 100 |  | 100 |  |
|  |  |  |  |  |  |  |  |  |  |  |  |  |
| Cancer |  |  |  |  |  |  |  |  |  |  |  |  |
| 0 (n=3,201) | 99.9 | [99.5,100.0] | 99.4 | [98.9,99.6] | 99.5 | [98.9,99.8] | 99.6 | [99.0,99.8] | 99.8 | [99.1,99.9] | 99.6 | [99.3,99.8] |
| 1 (n=12) | 0.1 | [0.0,0.5] | 0.6 | [0.4,1.1] | 0.5 | [0.2,1.1] | 0.4 | [0.2,1.0] | 0.2 | [0.1,0.9] | 0.4 | [0.2,0.7] |
| Total (n=3,213) | 100 |  | 100 |  | 100 |  | 100 |  | 100 |  | 100 |  |
|  |  |  |  |  |  |  |  |  |  |  |  |  |
| Epilepsy |  |  |  |  |  |  |  |  |  |  |  |  |
| 0 (n=3,201) | 99.7 | [99.3,99.9] | 99.5 | [99.1,99.8] | 99.3 | [98.6,99.7] | 99.9 | [99.4,100.0] | 99.6 | [98.8,99.8] | 99.6 | [99.3,99.8] |
| 1 (n=12) | 0.3 | [0.1,0.7] | 0.5 | [0.2,0.9] | 0.7 | [0.3,1.4] | 0.1 | [0.0,0.6] | 0.4 | [0.2,1.2] | 0.4 | [0.2,0.7] |
| Total (n=3,213) | 100 |  | 100 |  | 100 |  | 100 |  | 100 |  | 100 |  |
|  |  |  |  |  |  |  |  |  |  |  |  |  |
| CKD |  |  |  |  |  |  |  |  |  |  |  |  |
| 0 (n=3,208) | 99.7 | [99.3,99.9] | 99.9 | [99.6,100.0] | 100 |  | 99.8 | [99.3,99.9] | 99.8 | [99.1,99.9] | 99.8 | [99.6,99.9] |
| 1 (n=5) | 0.3 | [0.1,0.7] | 0.1 | [0.0,0.4] | 0 |  | 0.2 | [0.1,0.7] | 0.2 | [0.1,0.9] | 0.2 | [0.1,0.4] |
| Total (n=3,213) | 100 |  | 100 |  | 100 |  | 100 |  | 100 |  | 100 |  |

0= Disease absent, 1 Disease present

**Supplementary table 2 Prevalence of major dyads**

|  | overall | |
| --- | --- | --- |
| Dyads ( 2 chronic conditions) |  |  |
|  | col % | 95% ci |
| htn+diab (n=70) | 2.2 | [1.7,2.7] |
| htn+lbp (n=70) | 2.2 | [1.7,2.7] |
| Htn+arth (n=41) | 1.3 | [0.9,1.7] |
| Arth+lbp (n=32) | 1 | [0.7,1.4] |
| Diab+lbp (n=22) | 0.7 | [0.5,1.0] |
| Htn+hd (n=18) | 0.6 | [0.4,0.9] |
| Htn+thyroid (n=13) | 0.4 | [0.2,0.7] |
| Lbp+thyroid (n=14) | 0.4 | [0.3,0.7] |
| Htn+deafness (n=11) | 0.3 | [0.2,0.6] |
| Par+lbp (n=10) | 0.3 | [0.2,0.6] |

Arth:arthritis, can: Cancer; cld: chronic liver disease; copd: Chronic obstructive lung disease; diab: diabetes, gas: gastritis, htn: hypertension; lbp: lower back pain; par: paralysis/stroke; hd: heart disease; tb: Tuberculosis; thyroid: thyroid disease

**Supplementary table 3: Prevalence of major triad**

|  |  | |
| --- | --- | --- |
| Triad( ≥3 chronic conditions) |  |  |
|  | Col % | 95% CI |
| Htn+arth+lbp (n=20) | 0.6 | [0.4,1.0] |
| Htn+diab+lbp (n=14) | 0.4 | [0.3,0.7] |
| Htn+diab+hd (n=10) | 0.3 | [0.2,0.6] |
| Htn+lbp+thyroid (n=10) | 0.3 | [0.2,0.6] |

Arth:arthritis, can: Cancer; cld: chronic liver disease; copd: Chronic obstructive lung disease; diab: diabetes, gas: gastritis, htn: hypertension; lbp: lower back pain; par: paralysis/stroke; hd: heart disease; tb: Tuberculosis; thyroid: thyroid disease

**Supplementary table 4: Association of health outcomes across socio-demographic variables and number of morbidity**

| Variables | Disability | P value | Depression | P value | Low self-rated health | P value |
| --- | --- | --- | --- | --- | --- | --- |
|  | % [95%CI] |  | % [95%CI] |  | % [95%CI] |  |
| Age |  |  |  |  |  |  |
| 30-44years (Ref) |  |  |  |  |  |  |
| 45-59 years | 2.31 [0.71,7.56] | 0.2 | 1.03 [0.52,2.06] | 0.9 | 2.28 [1.89,2.74] | <0.001 |
| 60 years and above | 6.72 [2.01,22.42] | <0.001 | 1.01 [0.43,2.36] | 1.0 | 3.99 [3.10,5.12] | <0.001 |
|  |  |  |  |  |  |  |
| Gender |  |  |  |  |  |  |
| Male (Ref) |  |  |  |  |  |  |
| Female | 2.55 [0.65,10.02] | 0.2 | 6.17 [1.40,27.17] | <0.001 | 1.96 [1.49,2.59] | <0.001 |
|  |  |  |  |  |  |  |
| Marital status |  |  |  |  |  |  |
| Currently Married (Ref) |  |  |  |  |  |  |
| Widow/Widower | 1.57 [0.70,3.54] | 0.3 | * | * | 0.85 [0.63,1.15] | 0.3 |
| Never Married | 6.62 [0.80,54.98] | 0.1 | * | * | 0.80 [0.45,1.44] | 0.5 |
|  |  |  |  |  |  |  |
| Education |  |  |  |  |  |  |
| College graduation and above (Ref) | * | * | * |  |  |  |
| High school or secondary | * | * | * |  | 1.41 [0.93,2.14] | 0.1 |
| up to primary | * | * | * | * | 2.13 [1.35,3.36] | <0.001 |
|  |  |  |  |  |  |  |
| Occupation |  |  |  |  |  |  |
| Professional/Medium and big business owner | 1.00 [0.00,0.00] | <0.001 | * | * | 1.00 [0.00,0.00] | <0.001 |
| Skilled labourer/small business | 0.46 [0.03,7.17] | 0.6 | * | * | 0.89 [0.59,1.35] | 0.6 |
| Unskilled/semiskilled | 0.44 [0.05,3.92] | 0.5 | * | * | 0.95 [0.64,1.39] | 0.8 |
| Homemaker | 0.70 [0.06,7.64] | 0.8 | * | * | 1.35 [0.90,2.03] | 0.2 |
|  |  |  |  |  |  |  |
| Wealth Index |  |  |  |  |  |  |
| 1 (Ref) |  |  |  |  |  |  |
| 2 | 1.34 [0.59,3.02] | 0.5 | 0.56 [0.29,1.09] | 0.1 | 0.75 [0.61,0.92] | <0.001 |
| 3 | 0.83 [0.35,2.00] | 0.7 | 0.23 [0.09,0.62] | <0.001 | 0.71 [0.57,0.88] | <0.001 |
|  |  |  |  |  |  |  |
| Physical activity |  |  |  |  |  |  |
| Yes (Ref) |  |  |  |  |  |  |
| No | 2.30 [1.09,4.85] | <0.001 | 1.43 [0.66,3.08] | 0.4 | 1.10 [0.86,1.41] | 0.4 |
|  |  |  |  |  |  |  |
| Ever alcohol use |  |  |  |  |  |  |
| Yes |  |  |  |  |  |  |
| No | 1.21 [0.29,5.06] | 0.8 | 0.43 [0.07,2.56] | 0.4 | 1.03 [0.80,1.32] | 0.8 |
|  |  |  |  |  |  |  |
| Ever tobacco use |  |  |  |  |  |  |
| Yes |  |  |  |  |  |  |
| No | 1.00 [0.20,4.95] | 1.0 | 1.25 [0.25,6.19] | 0.8 | 0.67 [0.50,0.89] | <0.001 |
|  |  |  |  |  |  |  |
| BMI |  |  |  |  |  |  |
| Under-weight |  |  |  |  |  |  |
| Normal | 0.32 [0.08,1.25] | 0.1 | 0.57 [0.17,1.92] | 0.4 | 0.95 [0.61,1.48] | 0.8 |
| Overweight | 0.19 [0.03,1.04] | 0.1 | 0.91 [0.27,3.08] | 0.9 | 0.88 [0.56,1.40] | 0.6 |
| Obese | 0.54 [0.17,1.70] | 0.3 | 0.69 [0.23,2.09] | 0.5 | 1.06 [0.69,1.62] | 0.8 |
|  |  |  |  |  |  |  |
| No of morbidities |  |  |  |  |  |  |
| 0 (Ref) |  |  |  |  |  |  |
| 1 | 1.84 [0.8- 4.5] | 0.2 | 1.14 [0.5-2.4] | 0.7 | 1.4[1.2-1.7] | <0.001 |
| 2 | 2.33 [0.9-6.1] | 0.1 | 2.16 [0.9-5.0] | 0.1 | 3.39 [2.5-4.7] | <0.001 |
| ≥3 | 5.72 [2.1-15.4] | <0.001 | 4.27 [1.6-11.1] | <0.001 | 5.09[3.0-8.7] | <0.001 |

* Dropped from analysis as there was no participant in one of the category of these variables

**Supplementary table 5: Regression analysis to estimate association of multimorbidity weighted index across socio-demographic variables**

|  | Multimorbidity weighted index | |
| --- | --- | --- |
| Variables | AOR ( 95% CI) | p value |
| Age |  |  |
| 30-44years (Ref) |  |  |
| 45-59 years | -0.66 [-0.81,-0.50] | <0.001 |
| 60 years and above | -1.21 [-1.45,-0.98] | <0.001 |
|  |  |  |
| Gender |  |  |
| Male (Ref) |  |  |
| Female | -0.02 [-0.29,0.25] | 0.88 |
|  |  |  |
| Marital status |  |  |
| Currently Married | -0.32 [-0.67,0.03] | 0.07 |
| Widow/Widower | -0.31 [-0.76,0.14] | 0.18 |
| Never Married (Ref) |  |  |
|  |  |  |
| Education |  |  |
| College graduation and above (Ref) |  |  |
| High school or secondary | -0.03 [-0.39,0.33] | 0.89 |
| up to primary | -0.26 [-0.67,0.15] | 0.21 |
|  |  |  |
| Occupation |  |  |
| Professional/Medium and big business owner | 0.56 [0.17,0.94] | 0.01 |
| Skilled labourer/small business | 0.73 [0.40,1.07] | <0.001 |
| Unskilled/semiskilled | 0.81 [0.57,1.04] | <0.001 |
| Homemaker (Ref) |  |  |
|  |  |  |
| Wealth Index |  |  |
| 1 (Ref) |  |  |
| 2 | -0.24 [-0.43,-0.06] | 0.01 |
| 3 | -0.34 [-0.53,-0.14] | <0.001 |
|  |  |  |
| Physical activity |  |  |
| Yes | -0.27 [-0.50,-0.04] | 0.02 |
| No (Ref) |  |  |
|  |  |  |
| Ever alcohol use |  |  |
| Yes | -0.17 [-0.40,0.07] | 0.16 |
| No (Ref) |  |  |
|  |  |  |
| Ever tobacco use |  |  |
| Yes | -0.10 [-0.37,0.17] | 0.46 |
| No (Ref) |  |  |
|  |  |  |
| BMI |  |  |
| Under-weight | 0.18 [-0.20,0.56] | 0.35 |
| Normal (Ref) |  |  |
| Overweight | -0.01 [-0.25,0.23] | 0.91 |
| Obese | -0.33 [-0.52,-0.13] | <0.001 |
